# A 10-year prognostic model for patients with suspected angina attending a chest pain clinic

**DOI:** 10.1136/heartjnl-2015-308994

**Published:** 2016-02-29

**Authors:** Neha Sekhri, Pablo Perel, Tim Clayton, Gene S Feder, Harry Hemingway, Adam Timmis

**Affiliations:** 1 Department of Cardiology, Barts Heart Centre, London, UK; 2 London School of Hygiene and Tropical Medicine, London, UK; 3 Centre for Academic Primary Care, School of Social and Community Medicine, University of Bristol, Bristol, UK; 4 Farr Institute of Health Informatics Research at London, London, UK; 5 Department of Epidemiology and Public Health, University College London, London, UK; 6 NIHR Cardiovascular Biomedical Research Unit, Bart's Heart Centre, London, UK

## Abstract

**Background and objective:**

Diagnostic models used in the management of suspected angina provide no explicit information about prognosis. We present a new prognostic model of 10-year coronary mortality in patients presenting for the first time with suspected angina to complement the Diamond-Forrester diagnostic model of disease probability.

**Methods and results:**

A multicentre cohort of 8762 patients with suspected angina was followed up for a median of 10 years during which 233 coronary deaths were observed. Developmental (n=4412) and validation (n=4350) prognostic models based on clinical data available at first presentation showed good performance with close agreement and the final model utilised all 8762 patients to maximise power. The prognostic model showed strong associations with coronary mortality for age, sex, chest pain typicality, smoking status, diabetes, pulse rate, and ECG findings. Model discrimination was good (C statistic 0.83), patients in the highest risk quarter accounting for 173 coronary deaths (10-year risk of death: 8.7%) compared with a total of 60 deaths in the three lower risk quarters. When the model was simplified to incorporate only Diamond-Forrester factors (age, sex and character of symptoms) it underestimated coronary mortality risk, particularly in patients with reversible risk factors.

**Conclusions:**

For the first time in patients with suspected angina, a prognostic model is presented based on simple clinical factors available at the initial cardiological assessment. The model discriminated powerfully between patients at high risk and lower risk of coronary death during 10-year follow-up. Clinical utility was reflected in the prognostic value it added to the updated Diamond-Forrester diagnostic model of disease probability.

## Introduction

In the patient with stable chest pain, the diagnosis of coronary artery disease (CAD) is a probability judgement based on clinical presentation and disease prevalence in the population group to which the patient belongs. Quantitative analysis of disease probability was provided by Diamond and Forrester based on patient age, gender and typicality of symptoms.^1^ The Diamond-Forrester estimate of disease probability, its recent update^2^ and its modification in the Duke clinical score^3^ lie at the heart of contemporary guidelines for the management of patients with suspected angina.^4–6^ However, these diagnostic models provide no explicit information about prognosis and many patients diagnosed with atypical or non-cardiac chest pain based on low estimates of disease probability experience coronary events during follow-up.^7^ The importance of prognostic assessment is emphasised in stable coronary disease guidelines,^5^ ^8^ but prognostic models have not previously been developed in patients with suspected angina among whom outcomes are likely to vary considerably according to the presence and severity of CAD. A prognostic model to identify those high-risk patients who fall through the diagnostic net would provide an opportunity for treatment to protect against myocardial infarction and coronary death.^9^

In the present study we have examined associations between clinical variables available at first consultation and 10-year coronary mortality in a large cohort of patients presenting for cardiac assessment of previously undiagnosed chest pain. The main aim was to develop a new prognostic model of the 10-year coronary mortality risk in patients with suspected angina to complement the Diamond-Forrester diagnostic model of disease probability in their work-up and further management.

## Methods

### Patients

The patient population has been previously described.^7^ In brief, we included consecutive patients attending six UK chest pain clinics (Newham, Oldchurch, Kingston, Blackburn, Manchester, Burnley). The purpose of the clinics was to identify patients with angina to initiate appropriate treatment, including secondary prevention with aspirin and statins. Data on 11 082 patients were electronically recorded from 2 January 1996 to 31 December 2002 using identical databases, details of which have been reported previously.^10^ We excluded re-attendances during the study period (n = 448), patients without chest pain (n = 291), patients diagnosed with acute coronary syndromes on the day of visit (n = 246), patients who reported previously diagnosed coronary heart disease or revascularisation procedures (n = 579), patients for whom a diagnosis was either not entered (n = 132) or not identified as angina or non-cardiac chest pain (n = 83), those with missing data on key explanatory variables (n=501), and those who were not traced by the national death registry (n = 40).^11^ The remaining 8762 patients with complete data and follow-up constituted the study group.

### Data collection

Clinical data were systematically recorded at the time of the initial consultation in a purpose-built electronic database that was utilised across the six study centres. The data included age, sex, ethnicity, clinical descriptors of chest pain (duration of symptoms before attendance, character, site and radiation of chest pain, duration of an episode, precipitating factors and relief with glyceryl trinitrate), smoking status, history of hypertension and diabetes, pulse rate, and systolic blood pressure. Twelve-lead resting ECGs were recorded as normal or abnormal, respectively, depending on assessment of rhythm, conduction, and the absence or presence of regional ST segment or T wave changes, left ventricular hypertrophy, and Q waves. Fields for all these clinical and ECG findings were included in the database and were populated by the attending clinicians during consultation in the chest pain clinic. Clinicians were also invited to enter into the electronic record their assessment of the patient's chest pain as being ‘typical’, ‘atypical’ or ‘non-anginal’.

### Follow-up

Patients were flagged for mortality with the Office for National Statistics (to 31 December 2011).^11^ Successful matching was achieved in 99.5% of the cohort. Causes of death were defined by the WHO International Classification of Diseases (ICD-10 codes). Information about non-fatal events was unavailable because flagging of our patients with the NHS-wide clearing system had terminated in 2003.

### Main outcome measures

The primary end point was death due to coronary heart disease (ICD-10 I20–I25). The secondary end point was cardiovascular death (ICD-10 I00–I99).

### Bias

Bias in patient selection and data collection was minimised by including consecutive patients attending for the first time with suspected angina and by utilising a standardised electronic database across all study centres. Bias due to the variable demographic characteristics of the clinic populations was minimised by including a mixture of urban and suburban centres in this multicentre study.

### Ethical approval

Ethical approval was obtained from the multiregional ethics committee (MREC/02/04/095). Permission was given by the National Patient Information Advisory Group to link anonymised datasets without individual patient consent.^12^

### Statistical analysis

To derive the new prognostic model to predict coronary death we initially used data from the Newham centre (n=4412) as the development dataset because it comprised approximately half of all patients enrolled. Validation was assessed with data from the other five centres (n=4350) by applying the coefficients of the model from the development dataset to the validation dataset. Overall rates and rates by categories of the candidate predictors were calculated. For all variables of interest, data were complete or in two cases >99% complete (table 1). Cox proportional hazard models (complete case analysis) were used to estimate the univariable and multivariable associations of the predictor variables with coronary death. Candidate variables included demographics, cardiovascular disease risk factors, chest pain characteristics, and ECG findings. We included these variables because they have been reported as predictors in previous prognostic models in related populations.^13–15^ Predictor variables independently associated with coronary disease mortality were identified using a manual forward stepwise approach rather than an automated stepwise procedure to allow for clinical judgement and for variables not at first meeting the criterion for inclusion in the final multivariate model to be later reconsidered. Smoking, for example, was forced into the final model based on its well-established association with coronary mortality. There are several measures to assess characteristics of chest pain. It was decided a priori that the symptoms of chest pain (typical, atypical or non-cardiac chest pain) should be the assessment included as it is easily measured without the need for further diagnosis and is the measure included in previous scores. The linear relationship of quantitative variables was considered and grouped into appropriate categories where necessary or for ease of interpretation. The risk of dying within 10 years from a coronary cause was calculated for each individual using Kaplan-Meier estimates to allow that a number of individuals had <10 years of follow-up (but >9 years). Individuals were then divided into fourths of risk based on the quartile cut-points so that there were 25% of patients in each risk group. The Kaplan-Meier survival curves were then plotted for each of these groups.

**Table 1:** Univariable associations with coronary death: all patients

	Total	Deaths	Rate*	HR (95% CI)	p Value
All patients	8762	233	2.9		
Age (per 10 years)	8762	233		2.46 (2.19 to 2.76)	<0.0001
Age group					
<50	3341	22	0.68	1	
50 to <55	1151	17	1.54	2.27 (1.20 to 4.27)	0.011
55 to <60	1163	21	1.92	2.82 (1.55 to 5.12)	0.0007
60 to <65	1040	30	3.13	4.58 (2.64 to 7.94)	<0.0001
65 to <70	881	29	3.66	5.33 (3.06 to 9.28)	<0.0001
70 to <75	645	46	8.24	11.94 (7.19 to 19.85)	<0.0001
75 to <80	356	35	12.63	18.05 (10.59 to 30.77)	<0.0001
80+	185	33	26.12	36.65 (21.35 to 62.91)	<0.0001
Sex					
Female	4168	80	2.04	1	
Male	4594	153	3.61	1.76 (1.34 to 2.31)	<0.0001
Ethnicity					
Other	2977	46	1.61	1	
White	5785	187	3.54	2.18 (1.58 to 3.00)	<0.0001
Pulse rate (per 10 bpm)	8762	233		1.21 (1.10–1.33)	0.0001
SBP (10 mm Hg)	8762	233		1.20 (1.13–1.27)	<0.0001
DBP (10 mm Hg)	8711	231		1.15 (1.03–1.29)	0.014
Current smoker					
No	6667	174	2.80	1	
Yes	2095	59	3.06	1.09 (0.81 to 1.47)	0.55
Hypertension					
No	5717	118	2.20	1	
Yes	3045	115	4.14	1.87 (1.45 to 2.42)	<0.0001
Diabetes					
No	7844	178	2.43	1	
Yes	918	55	6.63	2.71 (2.00 to 3.67)	<0.0001
Family history of CAD					
No	5851	161	2.98	1	
Yes	2880	70	2.58	0.87 (0.66 to 1.15)	0.33
Character symptoms					
Atypical	5079	93	1.95	1	
Typical	2002	115	6.44	3.28 (2.50 to 4.31)	<0.0001
Non-cardiac	1681	25	1.58	0.81 (0.52 to 1.26)	0.35
ECG normal					
Normal	7291	128	1.86	1	
Abnormal	1471	105	8.24	4.37 (3.38 to 5.66)	<0.0001

_*Per 1000 person-years.

CAD, coronary artery disease; DBP diastolic blood pressure; SBP, systolic blood pressure.

Agreement was very good between the development and validation datasets, indicating that any bias consequent on single site (Newham) model development was minimal. In addition, a separate model was produced in the validation dataset using the variables identified in the development dataset. There was close similarity in the associations of predictor variables with coronary disease mortality in the two models, and therefore in order to maximise power and the precision of the estimates, the prognostic model was finalised using all 8762 individuals. The performance of the model was assessed in terms of calibration and discrimination. Calibration was assessed graphically by plotting the observed outcomes compared with the predicted probabilities (by quartiles of risk) and using the Hosmer-Lemeshow goodness-of-fit test. Discrimination was estimated by calculating the area under the receiver operating curve (C-statistic).

Risk estimates of CAD were calculated according to the updated Diamond-Forrester diagnostic model proposed by Genders.^2^ The predicted risk of CAD was then tabulated by the observed mortality to see how well previously published models for CAD risk predicted actual 10-year mortality due to coronary heart disease. Additional analyses also tabulated predicted CAD risk by all-cause mortality and cardiovascular mortality.

### Model presentation: online risk calculator

**Figure 1:**
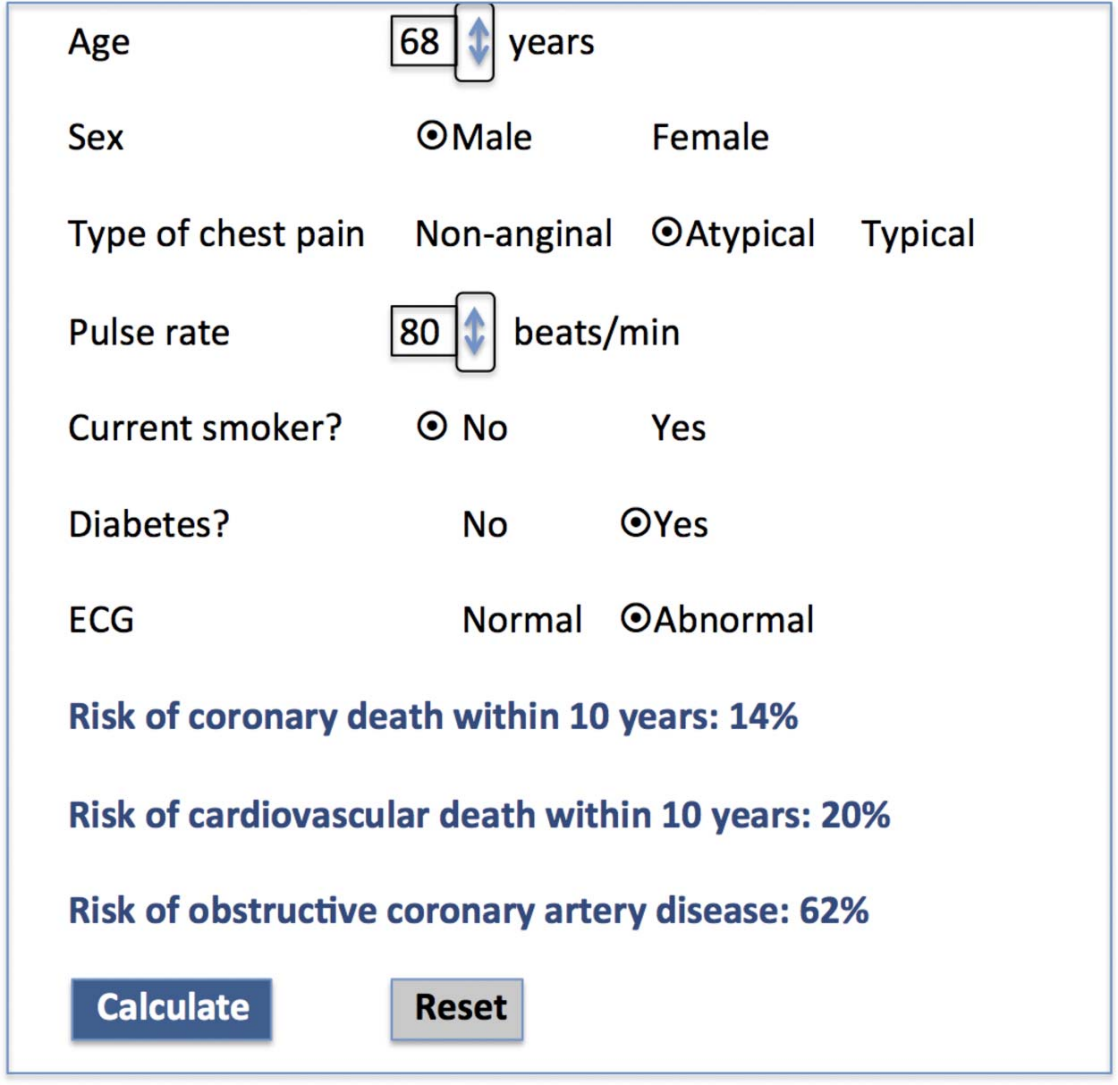
Diagrammatic screen shot of online prognosis in suspected angina (PISA) calculator for predicting the risks of coronary and cardiovascular mortality and the probability of obstructive coronary artery disease in patients with previously undiagnosed stable chest pain.

A prognosis in suspected angina (PISA) calculator utilising our model coefficients is available at https://www.sealedenvelope.com/trials/pisa/ allowing the estimated 10-year risks of coronary and cardiovascular mortality to be obtained for individual patients at the time of consultation (figure 1). By entering the predictor values the PISA mortality estimates are displayed and also the updated Diamond-Forrester estimates of disease probability.^2^

### Role of funders

Study funders acknowledged below played no role in the data collection, or its analysis and interpretation.

## Results

The 8762 patients were followed up for a median of 10 years (minimum 9 years). Two hundred and thirty-three coronary deaths were recorded, representing a rate of 2.9 deaths per 1000 patient years. Patients in the highest quarter of risk were older and more commonly male compared with patients in the lower risk quarters (see online supplementary table S1). The frequency of diabetes and typical angina symptoms increased across quarters of risk.

10.1136/heartjnl-2015-308994.supp2 Supplementary table 1 Characteristics by risk group

### Model development and validation

**Figure 2:**
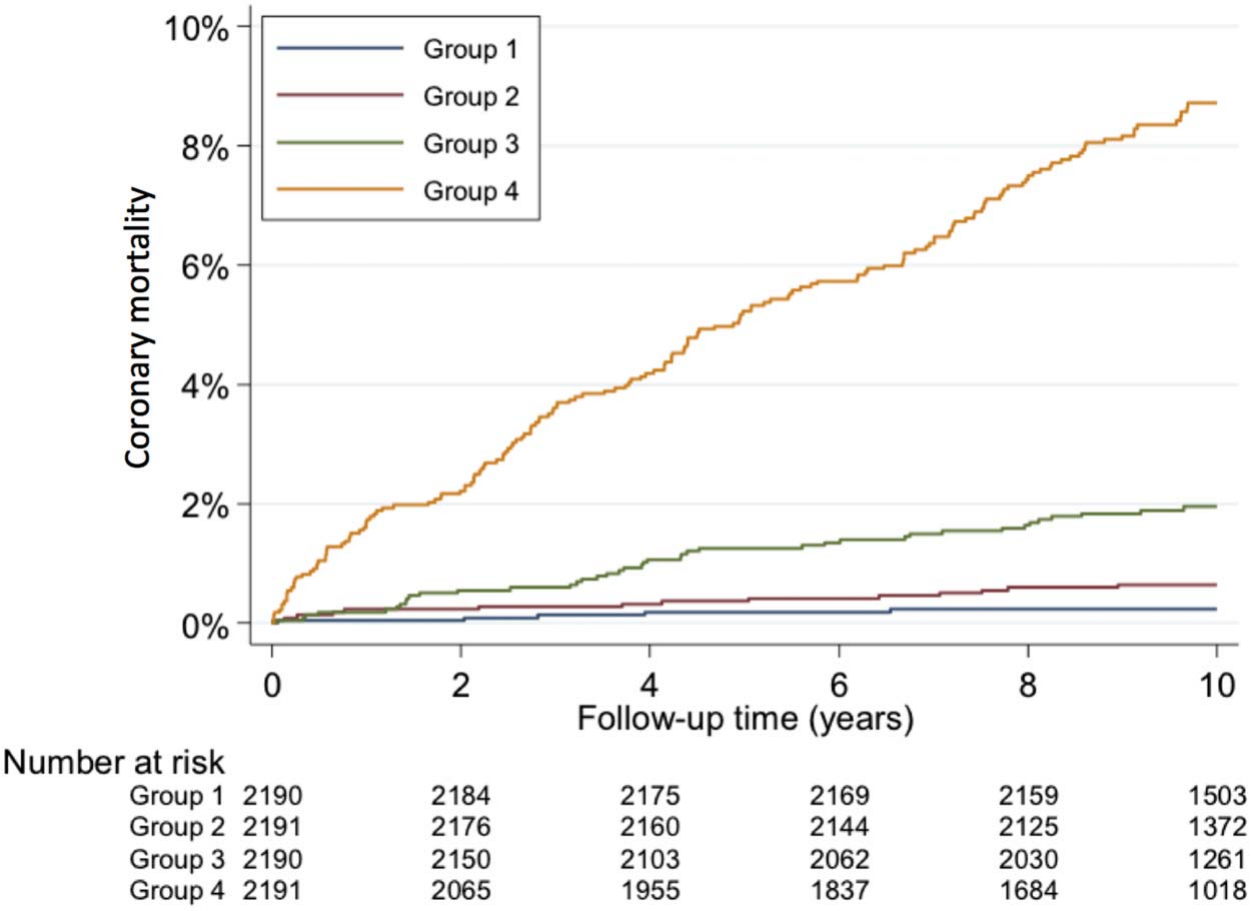
Kaplan-Meier cumulative coronary mortality by quarters of risk for the full prognostic model (based on table 2). There were 5, 14, 41, and 173 coronary deaths in risk groups 1 (lowest risk quarter) to 4 (highest risk quarter), respectively.

The model produced in the development dataset (see online supplementary table S2) and its external validation in the validation dataset (see online supplementary table S3) both showed good discrimination (c=0.84 and 0.82, respectively), and good calibration (see online supplementary figureS1). Because external validation was good and multivariate associations with coronary death were similar in both the developmental and validation datasets (see online supplementary tables S2 and S3), the final prognostic model was developed among all 8762 patients to maximise power. The final model, based on clinical factors available at the first presentation, showed strong associations with coronary disease mortality for age, sex, chest pain typicality, smoking status, diabetes, pulse rate, and ECG findings (tables 1 and 2). Discrimination (c=0.83) and calibration (table 2 and online supplementary figure S2) were strong, with patients in the highest quarter of risk accounting for 173 coronary deaths during follow-up (10 year risk of death:8.7%) compared with a total of 41, 14, and 5 deaths, respectively, in the lower risk quarters. Kaplan-Meier analysis confirmed wide and increasing separation of the survival curves for the highest risk quarter compared to the three lower risk quarters throughout the 10-year follow-up period (figure 2). Entry of the seven predictor variables into the online prognosis in stable angina (PISA) risk calculator at https://www.sealedenvelope.com/trials/pisa/ permits display of an individual patient's risk estimates of coronary and cardiovascular mortality and also the Diamond-Forrester diagnostic estimates of disease probability (figure 1).

10.1136/heartjnl-2015-308994.supp1 Supplementary figures

10.1136/heartjnl-2015-308994.supp3 Supplementary table 2 Multivariable associations with coronary death – NGH patients (n=4412, 105 coronary deaths)

10.1136/heartjnl-2015-308994.supp4 Supplementary table 3 Multivariable associations with coronary death – non-NGH patients (n=4350, 128 coronary deaths)

**Table 2:** Multivariable predictors of coronary death (n=8762, 233 coronary deaths)

Variable	HR (95% CI)	p Value
Age (per 10 years)	2.33 (2.05 to 2.65)	<0.0001
Sex		
Female	1	<0.0001
Male	1.91 (1.45 to 2.52)	
Character symptoms		
Atypical	1	0.0043
Typical	1.59 (1.20 to 2.12)	
Non-cardiac	1.05 (0.67 to 1.63)	
Pulse rate (per 10 bpm)	1.22 (1.13 to 1.33)	<0.0001
Current smoker		
No	1	0.0016
Yes	1.64 (1.21 to 2.23)	
Diabetes (y/n)		
No	1	<0.0001
Yes	1.99 (1.46 to 2.70)	
ECG normal		
Normal	1	<0.0001
Abnormal	1.96 (1.49 to 2.59)	

Harrell's C=0.83.

Adding hospital to the above model (non-Newham vs Newham) HR: 1.01 (95% CI 0.77 to 1.32), p=0.29.

### Added value of PISA model

Observed 10-year coronary mortality increased progressively with updated Diamond-Forrester estimates of disease probability ranging from 0.2% to 25.4% in groups with probability estimates of coronary disease <10% and >90%, respectively (table 3). Only in groups with a disease probability >30% was the 10-year coronary mortality >1%, representing an annualised mortality rate of only 0.1%. Similar patterns of increasing cardiovascular and all-cause mortality were observed as the predicted risk of coronary disease increased. However, when the PISA model was simplified to incorporate only those factors used in the updated Diamond-Forrester diagnostic model (age, sex, and character of symptoms), it caused it to underestimate the predicted coronary mortality risk compared with the full PISA model, particularly in patients with reversible risk factors (see online supplementary tables S4 and S5). For example, if the predictor variables are entered into the PISA online risk calculator for a 68-year-old man with atypical chest pain and diabetes who is a non-smoker with a heart rate of 80 beats/min and an abnormal ECG, the read-out for estimated 10-year coronary mortality is 14%, with a 62% probability of obstructive coronary disease (figure 1). If the simplified model is applied in the same patient, restricted to those factors used in the updated Diamond-Forrester diagnostic model (age, sex, character of symptoms), the coronary mortality risk is substantially underestimated at only 5%.

10.1136/heartjnl-2015-308994.supp5 Supplementary table 4 Simplified prognostic model. Predictors of 10 year cardiac mortality using only factors for calculating the updated Diamond-Forrester estimates of disease probability – all patients

10.1136/heartjnl-2015-308994.supp6 Supplementary table 5 Predicted quarters of risk for 10-year coronary mortality by simplified model and full model in 8762 patients

**Table 3:** Observed 10-year mortality by updated Diamond-Forrester estimates of coronary disease probability

Predicted risk group of CAD (%)	Total	Mean predicted risk of CAD (%)	Mortality*		
			Coronary	Cardiovascular	All-cause
<10	537	7.1	1 (0.2%)	5 (0.9%)	12 (2.3%)
10 to <30	3088	19.5	23 (0.8%)	51 (1.7%)	161 (5.3%)
30–60	3296	43.5	81 (2.6%)	124 (3.9%)	312 (9.6%)
>60–90	1753	74.2	111 (6.8%)	156 (9.6%)	387 (22.5%)
>90	88	91.2	17 (25.4%)	28 (39.1%)	52 (60.4%)

*Percentages from Kaplan-Meier estimate at 10 years.

CAD, coronary artery disease.

## Discussion

We have analysed long-term outcomes in 8762 patients presenting for the first time with suspected angina. A prognostic model is presented based on clinical factors routinely available at the initial cardiac assessment. The model discriminated powerfully between patients at high risk and at lower risk of coronary mortality during the 10-year follow-up period. Its potential clinical utility was reflected in the prognostic value it added to the updated Diamond-Forrester estimates of disease probability.^2^ To exploit clinical utility we have developed a user-friendly online PISA calculator that can be used in the consulting room to inform clinical decision-making.

International guidelines emphasise the importance of the clinical history in evaluating the patient with stable chest pain.^4–6^ However, while diagnostic models are widely used for assessing the probability of coronary disease, the only available prognostic models are those developed in populations with established disease.^13–15^ These are high-risk populations of little relevance to patients presenting for the first time with suspected angina, only a minority of whom have obstructive CAD to account for their symptoms.^16^ ^17^ Overall risk in such patients is low; the recent Scot-Heart trial, which recruited 9849 patients with suspected angina, reporting an estimated cardiovascular mortality of only 0.3% during a median 1.7 years of follow-up.^17^ Risk, however, is spread more widely than among patients with established coronary disease, and discrimination of the high risk minority from the lower risk majority, many of whom will have unobstructed coronary arteries, is central to clinical evaluation and management strategies. Guidelines recommend risk assessment based on the results of non-invasive tests^4–6^ even though the incremental value of such tests may be limited, the exercise ECG, for example, adding little to the diagnostic or prognostic information provided by the clinical assessment.^16^ ^18^ This has provoked calls for more effective methods of risk stratification in this group of patients in order that high-risk subgroups might be identified for more intensive investigation and treatment.^19^

The prognostic model developed in our study provides a ready means of risk-stratifying patients with suspected angina, being based on factors that are always available at the first clinical evaluation in the consulting room. Consideration of just seven factors—age, sex, typicality of symptoms, diabetes, smoking status, heart rate, ECG changes—permitted estimation of 10-year coronary mortality with excellent discrimination between patients in the highest and the lower risk quarters, as reflected in the 0.83 C statistic. The highest risk quarter accounted for >75% of coronary deaths during follow-up, the Kaplan-Meier estimate of 10-year mortality being close to 9% compared to <1% in the lowest risk quarter. The risk factors incorporated in the PISA model are well recognised, but in the present study their influence on coronary mortality in a chest pain clinic population has been quantified for the first time with the development of a desk-top PISA calculator for use in the clinical setting. Discrimination between high and low risk patients provided by the prognostic model identifies it as a potentially important clinical tool for selecting those patients with suspected angina who might benefit from more intensive investigation and treatment. Importantly, our data showed that prognosis inferred from the factors included in the updated Diamond-Forrester diagnostic model underestimated coronary mortality, particularly in patients with risk factors such as diabetes for whom chest pain presentations may be atypical, masking significant coronary mortality risk.^20^

Guidelines recommend that decisions about the further investigation of patients with suspected angina should be informed by probability estimates of CAD,^4–6^ but these estimates are often exaggerated in contemporary populations and updated models have been developed that have already penetrated international guidelines.^2^ ^16^ These updated models, however, like their predecessors, were developed in angiographic populations and it remains likely that they overestimate disease probability in the lower risk populations presenting for the first time with suspected angina. Thus, we found that only in groups with a Diamond-Forrester estimate of disease probability >30% was the observed 10-year coronary mortality >1%, representing an annualised mortality rate of only 0.1%. Even groups with an estimated probability of coronary disease of 60–90% (mean 74%) had an annualised coronary mortality rate of only 0.7%. This apparent mismatch between disease risk and mortality risk emphasises the importance of considering both diagnostic and prognostic indicators in patients with suspected angina in order that those at greatest risk might be identified.

The PISA web-based tool we developed has the potential to enhance the clinical assessment of patients with suspected angina by providing quantitative estimates of both disease probability, using the updated diagnostic model of Genders *et al*, and coronary and cardiovascular mortality using the prognostic model developed in our chest pain clinic population. The prognostic model was designed to identify high risk patients presenting for the first time with stable chest pain, and only those demographic and clinical factors consistently available at the first consultation were considered. Not considered, therefore, were factors that might later become available including the results of investigations such as non-invasive ischaemia tests and circulating lipid concentrations. For the same reason, treatment strategies adopted during the 10 years of follow-up were also not considered. Despite this, model performance was excellent based on just seven predictor variables which, when entered into the PISA web-based tool, provide a reliable estimate of 10-year coronary and cardiovascular mortality risk in patients with suspected angina. Use of the full model with entry of all seven predictor variables is important because prognostic assessment based simply on age, sex, and character of symptoms—the factors that populate the Diamond-Forrester model for estimating disease probability—takes no account of contributions made by risk factors, heart rate, and ECG findings, resulting in variable underestimation of mortality risk. Because data were collected across six different UK centres, the PISA risk estimates are likely to be generalisable, but further validation studies will be needed to confirm this.

Strengths of our study include the large multicentre patient population, the contemporaneous recording of chest pain characteristics and other clinical factors available at first consultation in a purpose built electronic registry, and the ascertainment of long-term cause-specific mortality through linkage with the UK national death registry. It was our main purpose to develop a prognostic model for utilisation at the first clinic attendance before instigation of further testing or specific treatments. Nevertheless, the potential for treatment and lifestyle changes to distort the risk prediction is a relevant consideration and emphasises the importance of measuring model discrimination over a prolonged period and validating the model in different populations. The fact that the model discriminated powerfully between risk groups during 10 years of follow-up, with comparable validity in two separate chest pain clinic populations, speaks to its utility independently of subsequent treatment and lifestyle change. Completion of the baseline data collection 12 years ago is a limitation, but allowed collection of the follow-up data and development of the long-term prognostic model that was the primary aim of this study. Other potential limitations relating to bias in patient selection, data recording, and catchment population characteristics were minimised by including consecutive patients with suspected angina, utilising a standardised database across study centres and recruiting from six different study centres in urban and suburban environments.

The broad spectrum of risk that characterises patients presenting with suspected angina challenges clinicians to identify the minority at high risk of coronary events. This can be achieved using the prognostic model and PISA online calculator developed in our study. The model is based on simple clinical factors available at the initial consultation and has the potential to complement diagnostic models of disease probability in identifying high-risk patients with suspected angina who merit more intensive investigation and treatment.

**Key messages**

**What is already known on this subject?**

- In patients with suspected angina, diagnostic models of disease probability lie at the heart of contemporary management guidelines.
- These diagnostic models provide no explicit information about prognosis.
- There are no prognostic models available in this patient population.

**What might this study add?**

- An internally validated prognostic model is presented for patients presenting for the first time with suspected angina.
- The model was developed using clinical factors routinely available at the initial cardiac assessment.
- The model discriminated powerfully between patients at high risk and at lower risk of coronary mortality during the 10-year follow-up period.

**How might this impact on clinical practice?**

- The prognostic model provides clinicians with the potential to identify those high-risk patients with suspected angina who fall through the diagnostic net.
- Clinical impact is reflected in the prognostic value the model adds to estimates of disease probability.
- To exploit clinical utility a user-friendly online prognosis in suspected angina calculator is provided to inform clinical decision-making in the consulting room.
