# Supplementary material for: A 10-year prognostic model for patients with suspected angina attending a chest pain clinic
Source: Heart. 2016 Feb 29;102(11):869–75. doi: 10.1136/heartjnl-2015-308994 (PMC4893090; doi:10.1136/heartjnl-2015-308994)
Supplement: Supplementary table 1 — Characteristics by risk group [file heartjnl-2015-308994supp_tableS1.pdf]

Table S1 Characteristics by risk group

	Total	Risk group			
		Group 1<br>n (%)	Group 2<br>n (%)	Group 3<br>n (%)	Group 4<br>n (%)
All patients	8762	2190	2191	2190	2191
CHD death					
No	8529	2185 (99.8%)	2177 (99.4%)	2149 (98.1%)	2018 (92.1%)
Yes	233	5 (0.2%)	14 (0.6%)	41 (1.9%)	173 (7.9%)
Hospital					
NGH	4412	1272 (58.1%)	1154 (52.7%)	1023 (46.7%)	963 (44.0%)
Non-NGH	4350	918 (41.9%)	1037 (47.3%)	1167 (53.3%)	1228 (56.0%)
Age at first visit					
Mean (SD)	54.7 (13.0)	39.9 (6.7)	50.5 (6.5)	59.1 (7.1)	69.5 (7.9)
Age group					
<50	3341	2045 (93.4%)	1032 (47.1%)	239 (10.9%)	25 (1.1%)
50-<55	1151	131 (6.0%)	582 (26.6%)	381 (17.4%)	57 (2.6%)
55-<60	1163	14 (0.6%)	418 (19.1%)	557 (25.4%)	174 (7.9%)
60-<65	1040	0 (0.0%)	142 (6.5%)	545 (24.9%)	353 (16.1%)
65-<70	881	0 (0.0%)	17 (0.8%)	328 (15.0%)	536 (24.5%)
70-<75	645	0 (0.0%)	0 (0.0%)	135 (6.2%)	510 (23.3%)
75-<80	356	0 (0.0%)	0 (0.0%)	5 (0.2%)	351 (16.0%)
80+	185	0 (0.0%)	0 (0.0%)	0 (0.0%)	185 (8.4%)
Sex					
Female	4168	1277 (58.3%)	1126 (51.4%)	1012 (46.2%)	753 (34.4%)
Male	4594	913 (41.7%)	1065 (48.6%)	1178 (53.8%)	1438 (65.6%)
Character symptoms					
Atypical	5079	1399 (63.9%)	1506 (68.7%)	1299 (59.3%)	875 (39.9%)
Typical	2002	67 (3.1%)	266 (12.1%)	576 (26.3%)	1093 (49.9%)
Non-cardiac	1681	724 (33.1%)	419 (19.1%)	315 (14.4%)	223 (10.2%)
Pulse rate (beats/min)					
Mean (SD)	76.7 (11.8)	74.8 (10.1)	76.3 (11.2)	76.9 (11.6)	78.8 (13.8)
Current smoker					
No	6667	1813 (82.8%)	1622 (74.0%)	1591 (72.6%)	1641 (74.9%)
Yes	2095	377 (17.2%)	569 (26.0%)	599 (27.4%)	550 (25.1%)
Diabetes (y/n)					
No	7844	2155 (98.4%)	2046 (93.4%)	1945 (88.8%)	1698 (77.5%)
Yes	918	35 (1.6%)	145 (6.6%)	245 (11.2%)	493 (22.5%)
ECG normal					
Normal	7291	2136 (97.5%)	2032 (92.7%)	1863 (85.1%)	1260 (57.5%)
Abnormal	1471	54 (2.5%)	159 (7.3%)	327 (14.9%)	931 (42.5%)
