# Supplementary material for: A 10-year prognostic model for patients with suspected angina attending a chest pain clinic
Source: Heart. 2016 Feb 29;102(11):869–75. doi: 10.1136/heartjnl-2015-308994 (PMC4893090; doi:10.1136/heartjnl-2015-308994)

Supplementary Figures

Figure S1: Observed vs predicted percentage of 10 year coronary death by quarter of risk – NGH patients (based on model in table S1)

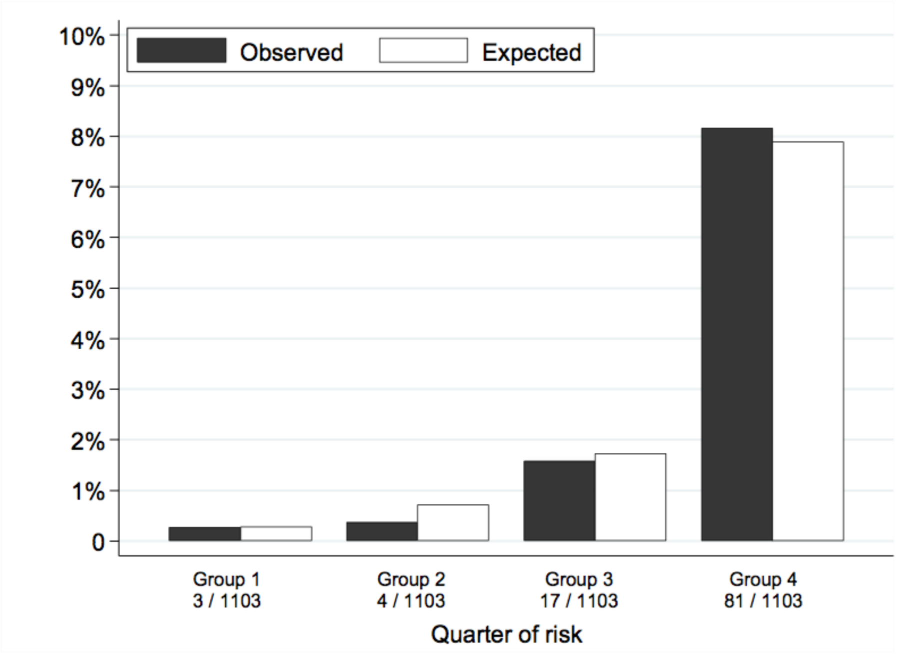

Figure S2 Observed vs predicted percentage of 10 year coronary death by quarter of risk in all patients

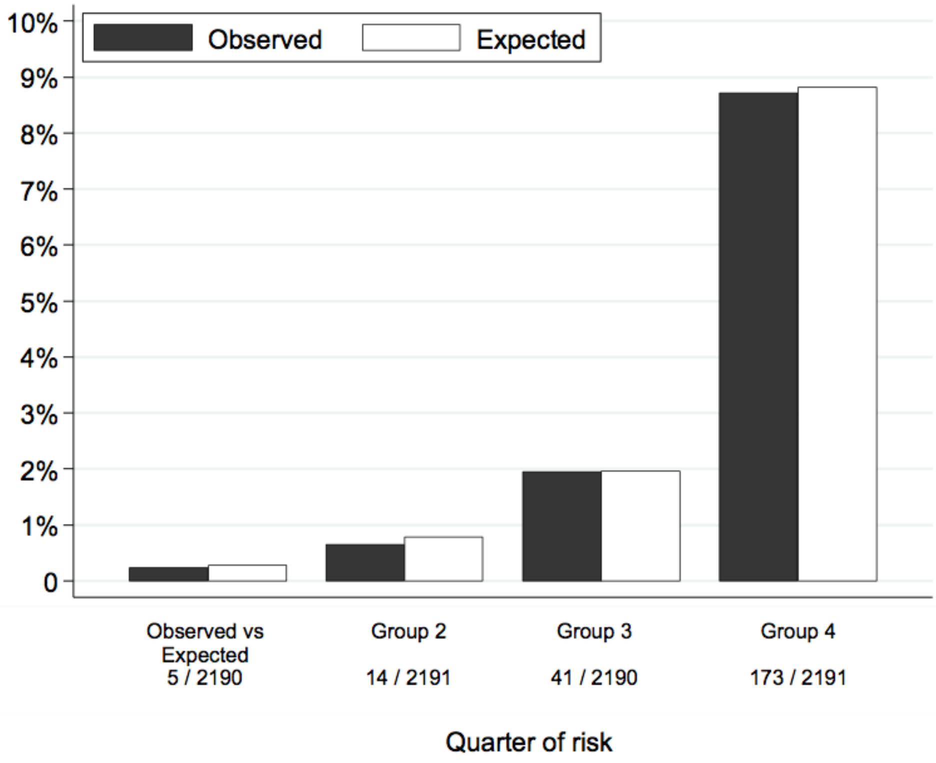

Supplement: Supplementary figures [file heartjnl-2015-308994supp_figures.pdf]
