# Supplementary material for: A 10-year prognostic model for patients with suspected angina attending a chest pain clinic
Source: Heart. 2016 Feb 29;102(11):869–75. doi: 10.1136/heartjnl-2015-308994 (PMC4893090; doi:10.1136/heartjnl-2015-308994)
Supplement: Supplementary table 2 — Multivariable associations with coronary death – NGH patients (n=4412, 105 coronary deaths) [file heartjnl-2015-308994supp_tableS2.pdf]

Table S2 Multivariable associations with coronary death – NGH patients (n=4412, 105 coronary deaths)

Variable	HR (95% CI)	
Age (per 10yr)	2.17 (1.82, 2.60)	p<0.0001
Sex		
Female	1	0.0004
Male	2.10 (1.39, 3.18)	
Character symptoms		
Atypical	1	0.12
Typical	1.55 (1.00, 2.41)	
Non-cardiac	1.03 (0.59, 1.81)	
Pulse rate (per 10 beats/min)	1.19 (1.05, 1.33)	0.0044
Current smoker		
No	1	0.081
Yes	1.50 (0.95, 2.35)	
Diabetes (y/n)		
No	1	0.0023
Yes	1.95 (1.27, 3.01)	
ECG normal		
Normal	1	0.0005
Abnormal	2.08 (1.37, 3.16)	

Harrell's C = 0.84

Non-NGH patients using above model: Harrell's C = 0.82
