# Supplementary material for: A 10-year prognostic model for patients with suspected angina attending a chest pain clinic
Source: Heart. 2016 Feb 29;102(11):869–75. doi: 10.1136/heartjnl-2015-308994 (PMC4893090; doi:10.1136/heartjnl-2015-308994)
Supplement: Supplementary table 3 — Multivariable associations with coronary death – non-NGH patients (n=4350, 128 coronary deaths) [file heartjnl-2015-308994supp_tableS3.pdf]

Table S3 Multivariable associations with coronary death – non-NGH patients (n=4350, 128 coronary deaths)

Variable	HR (95% CI)	
Age (per 10yr)	2.52 (2.09, 3.03)	p<0.0001
Sex		
Female	1	0.0025
Male	1.78 (1.23, 2.58)	
Character symptoms		
Atypical	1	0.0286
Typical	1.66 (1.13, 2.43)	
Non-cardiac	1.04 (0.49, 2.23)	
Pulse rate (per 10 beats/min)	1.28 (1.13, 1.46)	0.0002
Current smoker		
No	1	0.0047
Yes	1.85 (1.21, 2.83)	
Diabetes (y/n)		
No	1	0.0024
Yes	1.99 (1.28, 3.09)	
ECG normal		
Normal	1	0.0008
Abnormal	1.88 (1.30, 2.73)	
