# Supplementary material for: A 10-year prognostic model for patients with suspected angina attending a chest pain clinic
Source: Heart. 2016 Feb 29;102(11):869–75. doi: 10.1136/heartjnl-2015-308994 (PMC4893090; doi:10.1136/heartjnl-2015-308994)
Supplement: Supplementary table 4 — Simplified prognostic model. Predictors of 10 year cardiac mortality using only factors for calculating the updated Diamond-Forrester estimates of disease probability – all patients [file heartjnl-2015-308994supp_tableS4.pdf]

Table S4 Simplified prognostic model. Predictors of 10 year cardiac mortality using only factors for calculating the updated Diamond-Forrester estimates of disease probability – all patients

Variable	HR (95% CI)	
Age (per 10yr)	2.35 (2.09, 2.65)	p<0.0001
Sex		
Female	1	p<0.0001
Male	1.94 (1.48, 2.55)	
Character symptoms		
Non-cardiac	1	p<0.0001
Typical	1.70 (1.10, 2.64)	
Atypical	0.93 (0.60, 1.45)	

Harrell's C = 0.80
Global test of proportional hazards assumption, p=0.18
Adding hospital to the above model (non-NGH vs NGH) HR: 0.89 (95% CI 0.68 to 1.15), p=0.37
