# Supplementary material for: A 10-year prognostic model for patients with suspected angina attending a chest pain clinic
Source: Heart. 2016 Feb 29;102(11):869–75. doi: 10.1136/heartjnl-2015-308994 (PMC4893090; doi:10.1136/heartjnl-2015-308994)
Supplement: Supplementary table 5 — Predicted quarters of risk for 10-year coronary mortality by simplified model and full model in 8762 patients [file heartjnl-2015-308994supp_tableS5.pdf]

Table S5.Predicted quarters of risk for 10-year coronary mortality by simplified model and full model in 8762 patients

quarters (summary model)	quarters(full model) 1	2	3	4	Total
1	1,837	330	22	1	2,190
2	353	1,417	398	23	2,191
3	0	443	1,359	388	2,190
4	0	1	411	1,779	2,191
Total	2,190	2,191	2,190	2,191	8,762
